# Pathologia Indica, or the Anatomy of Indian Diseases

**Published:** 1845-07

**Authors:** 


### I. Pathologia Indica, or the Anatomy of Indian Diseases, Medical and Surgical, based upon Morbid Specimens from all Parts of India in the Museum of the Calcutta Medical College. Illustrated by Cases, &c. and Comments, Physiological, Practical, and Historical. By *Allan Webb*, B.M.S., &c. &c. Part I. Calcutta. Carey and Co. 1844.

### II. Transactions of the Medical and Physical Society of Bombay. Part VI. Bombay, 1843.

Dr. Webb's slender and most unpretending volume reflects the highest credit on its talented author. Within the compass of a hundred pages or so, he has managed to compress a large amount of curious physiological disquisition, as well as a good deal of valuable practical information. There is indeed a want of method and skilful arrangement in grouping many of the details that are brought forward; but this minor defect is amply compensated for by the importance of their character, and by the admirable scholarship that is often brought into requisition to bear and throw light upon them.

The first part of the work is taken up with a description of the preparations in the museum at Calcutta that are illustrative of the diseases of the Heart, Arteries, and Lungs: the descriptions being interspersed with reports of a good many cases, and with practical observations by Dr. Webb on the general pathology of these organs. The remaining portion of the volume is devoted to the more important—in reference to tropical disease—subject of the morbid conditions of the Liver and Spleen. This is the truly valuable part of the present work, and well merits an attentive perusal by all who practise in warm climates. We have little doubt that the observations and researches of our author on these points will be quoted and appealed to by subsequent writers on the pathology of Indian diseases.

The leading idea, we may premise, that pervades all Dr. Webb's remarks on the morbid conditions of the Spleen, is that this viscus is essentially and most intimately connected with the formation of the Blood; and the important inference, which he deduces from this position, is that whatever serves to derange the structure and functions of this organ, must tend to vitiate the condition of the circulating fluid. He points out a variety of considerations, anatomical as well as pathological, in support of this view; laying the chief emphasis upon the well-known and established fact, that a general Cachexy—indicated by such characters as Anæmia, Scorbutus, Dropsical effusions, tendency to passive hæmorrhage and to gangrene—is the usual concomitant of a diseased state of the Spleen. Whether he may not have sometimes carried his favourite doctrine a little too far, as, for example, when he says "that we can cure the diseased condition of the blood, by restoring the normal condition of the spleen," and such like statements, may very fairly, we think, be questioned; but that his facts and reasonings are well calculated to direct the attention of medical men to the (of recent years more especially) neglected subject of Splenic pathology, no one, who peruses his work with attention, will hesitate to admit. We propose in this article to follow him as closely as possible in his interesting researches, giving a condensed summary of what occupies about seventy pages in the original. Dr. Webb takes a rapid review of some of the most prominent opinions that have at different times been held as to the uses or functions of the Spleen. Like a good Catholic, he begins at the beginning.

Hippocrates regarded this viscus as the generator of the lymph or aqueous humour of the body; his words are: *Habet autem mulier et vir quatuor species humoris in corpore, e quibus morbi fiunt, qui non a violentiâ aliqua fiunt. Hæ sunt species; pituita, sanguis, bilis, et hydrops seu aqua: . . . . et sane sanguini fons est cor, pituitæ caput, aquæ splen.* According to this view of its physiology, the connexion of dropsical complaints with the existence of lesions of the Spleen was readily accounted for. It is a curious fact that in the Hindoo medical writings—the antiquity of which is so great that it has been very reasonably conjectured that much of the knowledge of the Greek physicians was derived from them—the function of the spleen is alleged to be the preparation of, and more especially the addition of the red globules to, the blood.* Galen was much less fortunate in his conjectures on this subject: he supposed that the office of the viscus was "to attract the viscid and melancholic juices generated in the Liver." The Arabian physicians adopted with certain modifications this view of the question; but it is not to be denied that, however unmeaning much of their physiology undoubtedly was, their observations on practical medicine are often most truthful and accurate. In the rare work of Hali Abbas, who lived in the eleventh century, we find it laid down, as a general position, that *whenever the spleen is enlarged, the body is emaciated;* the blood becoming at the same time thin and watery, and the extremities invariably cold. Avicenna has given a similar view of its functions and diseases. Dr. Webb remarks of his writings:—

"The main subject is first stated, as to the *office of the spleen* which is to 'free the blood from its heaviness and combustion.' This *sounds* strange, but its *sense* is precisely similar to what professor Schultz has stated, that the vesicles when burnt, or in his more modern phraseology 'when no longer capable of being acted upon by oxygen,' being 'old, *heavy* and useless,' they 'sink to the bottom,' in the vena portæ, and are excreted through the liver, or there reorganized. Liebig still more distinctly maintains this theory of combustion. Again, Avicenna attributes the natural and accidental black humours to this

---

* Dr. Webb gives a curious extract from the Sanscrit work "Sushruta," translated by *Baboo* Moodusoodun Gupta, the native demonstrator of anatomy in the Medical College at Calcutta. He remarks: "Not only is this extract valuable, because it proves that the Hindoos held this opinion, of chyle receiving its red colour in the spleen, and *there* becoming blood; the precise fact which I have all along been labouring to prove, but it is curious also as containing, perhaps the first distinct account in any language, of the existence of chyle; and both in this respect and in making the arteries the channels of its circulation, when perfected as blood, it is greatly in advance of the Physiology of the Greeks, who, with the exception of Galen, looked upon the arteries as air-vessels only."

combustion and heaviness. This idea is more definitely stated at p. 59 of this work. 'If the old deeply coloured vesicles are not excreted from the circulation as new ones form, the blood assumes a darker tint, and the portal system is congested.'" 196.

Hewson, at the close of the last century, endeavoured to shew that this viscus was connected, in some way or another, with the formation of the vesicular envelopes of the red globules;—a doctrine which has received not a little confirmation of late years by the researches of Professor Schultz of Berlin. This gentleman affirms that, "on a chemical examination of the portal blood, its *plasma* is found to be less in quantity, and more fluid, and to contain more colouring matter than that of ordinary venous blood;" and he adds that "in the vena portæ two things take place: 1, the old useless vesicles are taken out of the circulation; and 2, the debris or dead films of these vesicles are separated from the blood"—a doctrine not very dissimilar to that of old Hali Abbas, who expressly asserts that "it is the office of the spleen to clear off the muddiness and feculence (debris) of the blood." The opinion that the Spleen is mainly conducive to the preparation of the red globules of the blood has been adopted by several recent writers, as Bourgery, Donné, &c. The latter of these gentlemen has given the following description.

"The globulines are the product of the chyle, which is continually being added to the blood. Three or four of these unite together, and whilst circulating with the blood, receive an albuminous envelope. They thus form the white globules. The white globules once formed, change little by little their forms; become flattened, coloured; granular matter in their interior becomes homogeneous or dissolved, and they are thus transformed into the red or proper blood-globules. The blood-globules themselves have only a passing existence; they dissolve after a certain time, and constitute the so-called *liquor sanguinis.* He says that certain substances, as milk, are capable of being immediately transformed into the blood-globules, by being injected into the blood-vessels, *and he regards the spleen as the organ more especially charged with the important function of the manufacture of blood-globules.*" 164.

The argument, derived from the fact that the Spleen has occasionally been extirpated, not only from some of the lower animals but even from man himself, without any apparent injury to life, does not weigh for so much as has been supposed; for we well know that the living system is endowed with extraordinary powers of compensation. It is rather by watching the effects of disease, than by the performance of rude mutilating experiments, that we can hope to arrive at any rational conjectures on the point at issue. Now "it is a fact," says our talented author," that spleen disease in this country, whenever it affects the young, is always followed by a dangerously disordered state of health, uniformly characterized *by a want of red blood.* We may conclude therefore, that an uniform relation, as cause and effect, subsists between enlargement of the spleen and this state of the blood. For it is just as easy, in nine cases out of ten, where children are affected, to judge of the state of the spleen by the state of the palpebral conjunctiva, as to judge of the heart's action by the state of the pulse. If there be an increase and return of red vessels, the spleen is diminishing; if there be diminution of red vessels, the spleen is enlarging. This may apply more especially to the young, and I believe it does. But the spleen, and the thymus too, may not be the less essential, and important, should their chief function be to provide an increase of red particles of blood, commensurate with the rapid increase of all living organization in the young. It is therefore no logical conclusion to say the organ is of no use, because we cannot exactly discover, in what its use consists. The supra-renal capsules, the thymus gland, the thyroid and the spleen, may all have, at one time or other, an use, as well as the placenta; but which may not, at every period of life, be of the same necessity. Moreover, this view of its being designed to prepare red blood, seems borne out by other analogies; for women, who in this country have become completely chlorotic from long-continued spleen disease, nay even if it have resulted, as it does, in some young women, in general anasarca, yet will the whole of this disappear upon marriage, if followed by pregnancy, and sometimes it will never return. During this healthy activity of the system, the power of forming red blood returns, and the spleen regains its normal condition. Of this I have known some instances, besides the very curious case cited by M. Pinel, from the *Ephémérides des Curieux de la Nature*."

The anæmic condition of a person labouring under Spleen disease bears a very striking resemblance to that of a chlorotic patient; and we need not remind our readers how remarkably deficient in red globules the blood of such a patient invariably is.* It is to be remembered, also, that disease of this viscus is almost uniformly accompanied with some malady or another that is more or less intimately connected with a vitiated state of the circulating fluids; such, for example, as dropsical effusions, passive hæmorrhages from various parts, scurvy, and spontaneous gangrène. Our author concurs in the opinion of MM. Piorry, Nonat, and other French writers, that splenic enlargement is invariably present in (he does not say, as the former of these gentlemen does, is *the cause* of) intermittent fever.†

---

* For an account of Andral and Gavarret's experiments, vide the numbers of the Medico-Chirurgical Review for April, July, and October, 1841.

† "Morgagni has demonstrated that these enlarged spleens result from fever —See Epist. xvi. n. 6, Epist. xx. n. 2,30 and 51, Epist. xxxi. n. 2, especially Epist. xxxvi. n. 1—where the 'spleen weighed eight pounds.' The internal parts of this viscus did not seem to differ from their natural constitution.—Externally both the sanguiferous and lymphatic vessels appeared enlarged, *so that the lymphatics were discovered up and down through the coats of the spleen, and made a very beautiful appearance.*'

"His explanation of this morbid phenomenon—is full of sound reasoning and follows n. xviii., p. 200. 'For a viscus which is of itself lax, and cellular, and from which the return of the blood is slow, as it is to pass through the liver, before it enters the vena cava, is extremely liable to tumours, especially if that little share of strength which it has originally, being weakened by a disease of long continuance, and the blood being made inert and sluggish, some particles are left therein, which ought either to be corrected, or thrown out of the body.'

"'For the sluggish motion of the blood being increased for these reasons, while, like muddy water beside its channel, it is diverted into the cells of the spleen, it of course deposits therein whatever corpuscles it may contain, which

"Of this fact, in paludal countries, we have abundant proof, in the classical work of M. Monfalcon. Indeed M. Piorry has also stated, that 'the frequency of hypertrophy of the spleen, in these fevers, is so great, that it was found to be present in 154 out of 161 cases.' I have observed it in every part of our dominions in the east. In India from the Sutledge to the Burampooter; from Cape Comorin to the Hymalaya; nay, even through Burmah, and the Malay peninsula, Penang, and Singapore. In Calcutta it would be difficult to assemble half a dozen coolies without some of them, (perhaps half,) bearing the mark of the branding, which is so favourite a mode of treatment with natives;—and is derived from the Greeks and Arabs. At the annual invaliding committee also, among the gallant fellows who fought in China, I observed but too many instances of spleen disease, rendering the sufferers more helpless and miserable than their wounded comrades." 166.

Dr. Webb points out the inconsistency of not alluding to *spleen disease* in statistical medical reports (more especially in those of tropical maladies), while so prominent an importance is invariably attached to *liver disease.* "Can there be a doubt," says he, "that the spleen is as liable to organic derangement as the liver ?. . . . . . I verily believe that its morbid influence, although more slow, is equally sure." We may regard it as a fact beyond all dispute that, in almost every case of tropical fever, the liver and spleen —those two important organs of sanguification—are more or less seriously deranged in structure. Read what Major Tulloch says of the troops that returned from Arracan :

"The appearance of the 44th and 54th Regiments, when they reached their respective Presidencies, exhibited a melancholy instance of the baneful effect of this climate, on the European constitution. Almost every soldier had immediately to be placed under hospital treatment: their countenances are described as of a dull saffron hue, their lips pale, their features swollen and œdematous, their abdomens tumid, and their whole persons apparently enlarged in bulk; *all were labouring under diseased spleen or derangement of other important viscera,* quite beyond the control of medicine. The only chance of recovery rested on a speedy return to their native country, but numbers died before that remedy was available, and of those who left Arracan, scarcely one-half were alive at the end of twelve months." 167.

Dr. Webb tells us that, among the troops that serve in Bengal, spleen disease and *consequent scorbutus* are a very frequent cause of invaliding and of death.

So intimate, in his opinion, is the connection between certain morbid states of the spleen and the induction of genuine scurvy, that he seems to consider the latter as a necessary result of the former. His words are: "Now if the spleen be as intimately concerned in forming red blood, as I have with much labour endeavoured to prove that it is, we have only to stop its functions, and we shall then produce scurvy. Thus scurvy is induced by disease of the formative organs of the blood-globules, as effectually as it can be, by withholding the materiel from which blood is formed;

---

are heavier and more gross than the constitution can bear, and by this means, in part obstructing its own return, distends the cells of this viscus more and more. And the more the whole spleen is distended, by the distention of the cells, so much the weaker it is, and for that reason more liable to retain, in great measure, those fluids which afterwards flow into it.'"

—by feeding the animal man upon putrid meat, and bad grain, and washing down such a *pabulum vitæ* with stinking stagnant water. In either case the blood is not renewed; the body therefore feeds upon itself."

The most frequent of all the lesions of this organ, in tropical countries, appears to be enlargement with softening or *ramollissement* of its substance. The natives of India are very often afflicted in this manner; and in them the viscus is sometimes so much disorganised that it becomes lacerated by the mere effort of vomiting, by the slightest blow upon the side, or it may be without either straining or external violence. "Dr. Finch says, of the sepoys in Bengal, suffering from enlarged sleen—'occasionally, though seldom, it proves fatal by rupture, in which case death takes place suddenly, either at the commencement of the cold fit of an ague, or by even the slightest exertion of the invalid walking, it may be from his own charpoy (bed) to that of his comrades.'"

Mr. Greig has related the case of a patient who had had only two cold fits of an ague, and had walked out an hour before he died, yet in whom the spleen had become so distended and disorganised that it was found on dissection to have given way at several points, and to be so soft that it scarcely bore the slightest handling without breaking.

Our author lays it down as a general position—and the remark is one of great practical interest—that *ramollissement,* not of any one organ only but of the viscera generally, *is eminently the characteristic feature of Indian pathology.*

The substance of the spleen, as we have seen, is very often reduced to the consistence of a soft pulp or tarry fluid; the heart is sometimes so soft that it cannot bear its own weight without laceration; the lungs are not unfrequently in a similar condition; in the liver and intestines this morbid state is exceedingly common, and in the brain also it is of by no means rare occurrence.

Dr. Webb says that *the softened spleen seems sometimes to be evacuated by vomiting;* and he alludes to various cases in the way of illustration. Among these he cites one from the Observata Anatomica of Blasius (1674), in which we read that—in a patient who had been for some time afflicted with a severe pain at the pit of the stomach, accompanied with distressing vomiting—the spleen was found on dissection to be almost entirely wasted away; nothing remaining in its place save some dark-coloured globules of various sizes. In a case recorded in the Bombay Transactions, the softened spleen seemed to be evacuated by vomiting: its disorganised tissue being found in the vomited matter. The patient recovered. (Such a statement as this must be received with some hesitation.)

The following case (recorded by Dr. Green in the Transactions of the Calcutta Medical Society), where a direct communication was discovered to exist between the cavity of the stomach and a diseased spleen, may throw some light upon this point of pathology: it is moreover interesting in another point of view, as indicating the connection between splenic disease and a morbid state of the blood.

"ULCERATED COMMUNICATION BETWEEN STOMACH AND SPLEEN, SPLENIC SCORBUTUS.

"A seaman, *disease* fever, with enlarged spleen, occurring at sea.—*Symptoms,* fever; petechiæ, which had appeared at sea early in the disease; liquid bloody stools; enlarged spleen; swollen, spongy, ulcerated gums;—death on the 31st day after admission.

" *Post Mortem Examination.*

" *Head.*—Considerable effusion within the bag of the arachnoid, both superiorly and at the base of the brain; effusion between the arachnoid and pia mater: also largely within the ventricles.

" *Heart.*—In pericardium ℥j. of yellowish fluid—walls of right ventricle very thin, pale, and flabby; black liquid blood in all the cavities; in the right auricle was a separation of the blood into large whitish coagula, and a thin black liquid. *Liver.*—A small abscess, containing a thin pus, was found in it.

" *Stomach.*—A large ulcerated opening through its coats communicated with a large excavated ragged cavity in the spleen; the mucous membrane of the stomach pale; the edges of the ulcer thin, as if dissolved; a yellowish mucus within the stomach. *Spleen* enlarged, adherent closely to the stomach and to the abdominal parietes; the peritoneum, where adhered to, presented dark bloody spots of extravasation; the cavity or abscess in the spleen which appeared to be the result of ulceration—(an advanced condition of the state found in No. 2)—opened externally, on its surface, in several places. *Intestines.*—Mucous membrane of lower small bowels ulcerated, thickened, ecchymosed, and of a generally deep purple color; feculent contents. *Mesenteric glands* enlarged, hard. Lumbar glands hard, highly vascular, enlarged." 173.

In the majority of instances of fatal Hæmatemesis, no distinct rupture or laceration of the bloodvessels of the stomach is discoverable on dissection; the blood, it would seem, having oozed from numerous minute points. The most frequent concomitant appearances are the enlargement and other morbid states of the Liver and more particularly of the Spleen; and in short any abnormal condition, either of these or of the other abdominal viscera, that may impede or derange the circulation through the portal system.

One of the most interesting cases on record, in illustration of the pathological connection between a morbid condition of the Spleen and the occurrence of Hæmatemesis, is one related by Morgagni in his 36th Epistle.

A young man, of a naturally robust constitution, had been in a weak and ailing state of health for two years, when at length a large and hard tumour made its appearance in the left hypochondrium; it caused a sense of weight in the part, and great dyspnœa, especially upon exertion. One day he was suddenly seized with a copious vomiting of blood: this attack was accompanied with great prostration, fever, and an increase of the swelling. The vomiting and fever, however, quickly ceased, and the patient was then put upon a course of steel, strengthening diet, &c. After three months' continuance of this regime, the hardness, but not the size, of the tumour was much diminished; the countenance of the patient was still pallid and of a citron hue. Two or three months subsequently, the vomiting of blood returned, and a smart attack of fever supervened; this proved fatal in the second week. The following were the appearances found on dissection.

" The body being dissected, it was amazing what a small quantity of blood remained in all the vessels. And, for this reason, the viscera of the belly attracted the eyes by an unusual paleness, and almost whiteness, except the spleen, which preserved its natural colour; but this viscus was so much increased as to exceed the liver in bulk, and weighed four pounds and a half. Yet it was not harder than it generally is, except that on its convex surface, in one or two places, was contained, deep within its surface, a substance of a very solid nature, of the bigness of a large nut. In the trunk of the splenic vein, polypous concretions lay hid, which divided themselves, together with the branches of that vein, in a very elegant manner, even within the spleen. The liver was very pale, except that here and there it was marked with black spots. The gall-bladder, which was more pale than the liver, and even whitish, contained a little bile of a very dilute colour, a similar bile to which was not wanting in the fundus of the stomach. The other parts of the belly were sound.

"In the thorax the lungs on their anterior surface were pale; but on the back part they appeared inflamed, and were of a black colour, inclining to purple: but, when cut into, they discharged a great quantity of frothy serum. In the right ventricle of the heart was only a small polypous concretion; and in the left only a beginning thereof." Epist. xxxvi. n. 11.

Bartolinus, also, has related some interesting cases of Hæmatemesis connected with an enlarged, or otherwise morbid, state of the spleen. In one of these, when sixteen pints of blood had been lost in the course of three days, the tumour in the left hypochondrium subsided, and the patient ultimately recovered. (Hist. anat. var. 1657.)

Before we quit the subject of the pathology of the spleen, we must briefly advert to a morbid lesion of this viscus that one would think must be of very rare occurrence, seeing that Mr. Twining has not even alluded to it in his valuable paper on the Diseases of the Spleen,* and Pinel seems to doubt whether it has ever been observed†: we mean—

*Abscess of the Spleen.*—It is a singular fact that this malady has been so much overlooked by modern pathologists; for it seems to have been perfectly well known not only to the early fathers of medicine (Hippocrates, Ætius, Aretæus, &c.), but also to the physicians of the last century. One of the most curious instances that we know of is that related by Morgagni, in which an enormous abscess of the spleen seems to have been mistaken for an ascitic swelling. The operation of paracentesis had been twice performed, and a large quantity of pus had escaped from the puncture instead of water. The patient died on the day after the second operation; and on dissection the spleen was found to be so much enlarged, that it reached from the left hypochondrium down to the pubes, covering the viscera in front and on the sides. When opened, it was discovered to contain as much purulent matter as had been drawn off during life. After relating these particulars, Morgagni adds: "that pus indeed, as well as water, has been sometimes found in the spleen, I have taken notice to you before; but I do not remember that it has been ever found in such an immense quantity. In so great an extension of a viscus, which is not large, and a sensation of a fluctuating fluid, who would have blamed the spleen in particular? The seat of the pain, except in the beginning perhaps, could not have shown this."

In Van Swieten's Commentaries, also, there is an instructive case com-

---

* Transactions of the Calcutta Medical and Physical Society.
† Nosographie Philosophique, tom. ii, p. 501. 1818.

municated by De Haen, in which the Spleen was found to contain "a thick, white, and abundant matter; a great quantity of the like matter floating about in the abdomen."

Dr. Webb has related a case headed, "Abscess of spleen bursting into the intestines, with recovery of the patient." Such *may* have taken place; but the details scarcely warrant an absolute diagnosis. After recording another instance of (presumed) splenic abscess, he makes the following remarks in reference to it.

"How did the spleen so rapidly decrease in a single night?

"It seems to me probable that it had burst on the 18th, and that the matter, pus, and flakes in the pelvis had come from it. It had healed when the lymph was effused. The fact of its being felt immensely large, two days before the purulent purging came on, is known to my friend Dr. Sprenger who saw the case with me—that it could not be felt two days after is also known to him. There may have been also a metastasis to the colon—such is not an uncommon attendant upon splenic suppurations." 154.

In order that we may give a practical character to some of the facts which we have brought forward touching the pathology of the spleen, we shall here devote a page or two to the consideration of the *treatment* that promises most benefit in one form at least of hypertrophe of this viscus: viz: that which is associated with softening of its substance, and which occurs not unfrequently in agues and other febrile diseases of hot climates.

While fully admitting the intimate connexion between enlargement of the spleen and protracted intermittent fevers, we should be on our guard not to carry this pathological doctrine too far, as M. *Piorry* and some other continental writers have surely done when they have contended that the morbific miasm acts primarily on the spleen, and that the fever is the result or effect of the visceral lesion. The extravagance of many of that gentleman's assertions will prove their best corrective; for who can possibly believe with him that the spleen may be sensibly diminished in size, in the course of a single minute, by the administration of large doses of quinine; or that this remedy is useful in those cases *only* of typhoid and other fevers, in which there is some affection of the spleen present? Yet several medical men have (for a time, at least) given credit to these astounding assertions. M. Gouraud* for example tells us that, after having witnessed the practice of M. Piorry at La Pitié Hospital, he felt fully satisfied of the correctness of the statements now made; but that, having repeated them at the Hôtel Dieu in the service of M. Rostan, he found that the same results were obtained by the use of various other means besides quinine,—a circumstance that made him suspect the correctness of the interpretation given by the "grand plessimetriste." In prosecuting his enquiries a little further, he satisfied himself that there was no actual diminution of the spleen in those cases where the shrinking had seemed very rapidly to take place; but that this viscus was merely *refoulée* by the stomach that had become distended with wind, and that the greater resonance then perceived, on percussing the left hypochondriac region, was altogether attributable to this simple cause!

M. *Nonat*, one of the physicians of La Charité Hospital, has taken a

---

* Journal des Connoissances Medico-Chirurgicales, Dec. 1844.

much more sober view of the question under consideration than his brother of La Pitié. He expressly says: "We will not affirm that hypertrophy of the spleen produces intermittent fever; this has its *point de depart* in some unknown cause, whether of the nature of miasm or otherwise, and inappreciable in its nature; but certainly it cannot be denied that such hypertrophy is an occasional and predisposing cause, which keeps the system under the influence of this primitive agent."* M. Nonat, following the example of M. Bally, urges the importance of administering very large doses of the quinine,—from 12 to 40 or even 50 grains in the course of the twenty-four hours—in those cases of ague that are accompanied with enlargement of the spleen. Its use should not be discontinued, until the viscus has resumed its normal dimensions.

Dr. Levy, one of the physicians of the Val de Grace hospital, has satisfactorily shewn the superior efficacy of full doses of quinine over every other remedy in those dropsies that so frequently supervene upon agues, and which are so generally connected with enlargement of the spleen.† Dr. Bouyer‡ very justly remarks that it is not by depletory means that we should endeavour to reduce the size of the enlarged spleen, but rather by fortifying the general system, and promoting at the same time greater activity in the transit of the blood through the abdominal circulation. For this purpose the exhibition of bark and steel, the use of salt-water baths, of friction over the left hypochondrium, and the constant use of a flannel belt around the abdomen, and perhaps also an *emplastrum ammoniaci cum hydrargyro*—with which a scruple or half a drachm of quinine has been blended—to the affected side, are the most efficient remedies. He is unfavourable to the use of even local blood-letting, but thinks that dry cupping over the left hypochondriac region may be serviceable.

Dr. Morehead of Bombay has recently made some similar remarks upon this subject.§

"The complication," says he, "of an enlargement of the spleen is so common in cases of obstinate intermittent fever, and has been generally so much the object of special attention in practice, that it would be an omission here not to allude to it; yet I do not think that it calls for any great deviation from the general principles of treatment which I have endeavoured to lay down. In cases in which the vigour of the constitution has not been much impaired (and such are not very common), the mode of treatment of enlargement of the spleen by repeated small leechings, and the regular exhibition of aperients with sulphate of iron and quinine, as recommended by the late Mr. Twining of Calcutta, is, I think, very applicable; but I doubt its suitableness in cases, in which the cachexy has proceeded far, for in many of these it has seemed to me that the enlargement of the spleen gives place as the general health improves.|| In fact,

---

* Vide Medico-Chirurgical Review, No. 65, July, 1840.

† Vide Med.-Chir. Review, No. 66, Oct. 1840. ‡ Ibid. 69, July, 1841.

§ Transactions of the Medical and Physical Society of Bombay, No. 6. 1843.

|| In his recently-published *Traité Elementaire et Pratique de Pathologie Interne*, Vol. II., 1845, M. Grisolle, after remarking that enlargement of the spleen often rapidly subsides, when it is a concomitant of fever, observes: "if, on the other hand, this lesion is slowly developed and is not accompanied with pyrexial symptoms, and if the viscus is hard and voluminous, quinine, in whatever doses it may be administered, is utterly inefficacious. I have often seen M.

to improve the health by small doses of quinine with half-a-grain of sulphate of iron, and a few minims of diluted sulphuric acid thrice daily, to have recourse to pure air, appropriate light nourishing diet, and the use of the compound rhubarb pill as a laxative when required, has seemed the best mode of management."

Dr. Webb also bears testimony to the excellent effects of large doses of quinine in the anasarca and general cachexy that so generally accompany enlargement of the spleen. " I have," says he, " found half-a-drachm of quinine, given daily to a child four years old, remove the enlargement of the spleen in ten days. It has the advantage of being perfectly safe in the hands of any sensible person. There is no necessity for debarring children from play and exercise."

The injurious effects of depletory measures, in such cases as those we have been alluding to, are strikingly shewn in one of the reports of our author, headed, " Anasarca and Enlarged Spleen treated with Mercury." The treatment in this case seems to have been any thing but judicious; the result was fatal. We are tempted here to suggest a remark that has occurred to our minds more than once during the perusal of the present volume. Is it not the case that some of our East India brethren are in the habit of carrying the lowering system rather too far even in acute diseases, and of employing it in a good many instances where any form of sanguineous depletion cannot but be, we should think, hurtful? In the very next report to that which we have just quoted, the disease seems to have been one of remittent fever, of several days' standing, unaccompanied by any symptoms of decided local inflammation; yet the patient was bled to sixteen ounces, and fourteen grains of calomel were given immediately afterwards, followed too by purging draughts, and leeches to the temples, on the day preceding his death. The collapse was very rapid. On dissection, the lungs and heart were found to be pale and bloodless; the liver was enormously enlarged and gorged with blood; the spleen too was soft and congested with a thick tarry-looking blood. We may mention also that the blood, that had been drawn, was neither cupped nor buffed. Is it not therefore somewhat of a misnomer to designate such a case as this an " inflammatory congestion of the spleen?"

From the consideration of the Spleen we pass on to that of its neighbour, the Liver; our remarks, we may premise, being confined almost entirely to the subject of Hepatic Abscess. There is no disease, Dysentery alone excepted, so common in the East Indies, as inflammatory affections of the Liver—" the largest and most vascular organ in the body." If we bear in mind the enormous supply of blood to this viscus, we must at once perceive the urgent necessity of prompt and very copious blood-letting in all active inflammations of its parenchymatous substance. The shrinking or subsidence of the hyperæmic gland in the right hypochondrium after an ample bleeding is often very remarkable. According to

---

Chomel give 15 or 20 grains daily for eight or ten days without producing any effect on the splenic enlargement; and of late years I have exhibited it in still larger doses, and occasionally combined with steel; but I have never been able to determine the slightest diminution in the size of the swelling."

M. Piorry, "this diminution of the hepatic organ varies from one to three inches from above downwards in twenty-four hours." Dr. Murray, the late Deputy Inspector of H. M. Hospitals, lays down the following peremptory rules for the guidance of the Indian practitioner.

"In the treatment, chief reliance is to be placed on v. s. *carried to syncope* at the very onset, and repeated at intervals not exceeding twelve hours, till the acute symptoms yield; very rarely are more than three bleedings requisite: but in very dangerous cases, I have required to repeat it five times (*to syncope, or till the pain ceases*) before the disease was sufficiently subdued to render the patient safe. This rule I consider the only true criterion, in severe cases, by which we can be guided as to the quantity of blood the constitution will bear to lose, and the disease require to be taken. The effect of bleeding to sixty or fifty ounces at once, in the commencement, is most satisfactory in subduing alarmingly dangerous symptoms; and I never saw any unfavourable consequences from it. One small bleeding afterwards—still to fainting—or leeches, are in general found sufficient to remove any remaining acute morbid action." 109.

In unfavourable cases, the inflammatory action often passes very rapidly into the suppurative, and a large portion of the hepatic parenchyma becomes thereby broken down, within a short space of time, into a soft pulpy purulent mass, without any circumscribing sac of membranous deposit between it and the intact portion of the liver. We need scarcely add that such diffuse abscesses are always much more dangerous than those that are confined within a cyst. Absorption of the purulent matter cannot take place;* and, if an artificial opening be made into the abscess, "the admission of air," says our author, "into the undefended and disrupted tissues of the organ can only be followed by sphacelus and death;—whilst it is to be regretted that, during life, there exists no satisfactory indication of the state of the abscess in this respect, whether it be encysted or not."

It is the opinion of many experienced practitioners in India that *abscesses in the liver may and do remain stationary in many cases for years.* The only information that our author furnishes on this head is in these words:

* Mr. Webb quotes with deserved commendation the following most just and lucid remarks by Dr. Copland.

"The functions of the membrane lining abscesses are not confined to the containing and isolating the purulent matter, so as to prevent the contamination of the adjoining structures. Owing to the absorption and exhalation proceeding in its surface, the contained fluid is continually renewed, its qualities are modified, and its decomposition prevented. It is not altogether removed from the influence of life, but participates in the vitality of the surrounding textures, all fluids accumulated in organised parts do, though in a feeble and obscure degree. M. Dupuytren remarks, that it is through the medium of this living envelope that the matter contained in abscesses is augmented and diminished in quantity; is thickened, or rendered more fluid; or is occasionally changed by substances absorbed or injected into the circulation. It is because the cysts of abscesses are connected by an intimate sympathy with the chief centres of vitality that the excitation of the more important viscera affects them in so marked a manner; and that remedies, judiciously applied to these viscera, often tend to promote the absorption of the matter they contain."—*Dictionary of Pract. Med., p.* 13.

"Such is my own opinion, and that of other surgeons in this country, both civil and military, who have had much greater experience. Our worthy president of the Medical and Physical Society, Mr. Egerton, has repeatedly expressed himself to this effect; so also has Dr. Murray, whom I have already quoted, nor is such a supposition, in any wise, inconsistent with what is observed of encysted abscesses located elsewhere, nor with the opinion of Dr. Copland, of their contents participating in a modified vitality, dependent upon the adjacent structures." 111.

The treatment of Hepatic Abscess is a subject of the highest interest to the East India practitioner, as the disease is one of such frequent occurrence in tropical countries. In not a few instances, Nature effects a cure either by eliminating the purulent matter in the way of absorption, or by discharging it through some outlet, as the bowels or even the lungs; and the patient will occasionally recover from so serious a lesion. It is rare, however, that things terminate so favourably as they did in the following "case of abscess in the liver spontaneously evacuated by the air-passages and bowels." A gentleman had two attacks of acute hepatitis, one following the other, within the space of a month. On the 13th day after the commencement of the second invasion, "a sudden change for the better, in the character of the symptoms, took place. He felt himself all at once relieved, and was sensible of something having given way within him. On examining his motions next day, a very considerable quantity of purulent matter was discerned in them, and in those he passed for several days after, which sufficiently warranted the opinion that had been held, of an abscess having been formed in the liver. For ten or twelve days after this, he improved considerably, when another return of the symptoms took place. The same remedies were employed as before, together with anodyne fomentations, with the same want of success; he got daily worse; and serious apprehensions regarding his recovery were entertained—when, on the 4th of October, he experienced another sudden change for the better. But this abscess being higher situated in the organ than the former one, burst into the *thorax* instead of the *colon*, and the matter was discharged by expectoration. Ever since he has continued to get better; and nothing further was required than a careful attention to the state of the bowels,—keeping them open by mild aperients and emollient clysters,—improving the strength generally by demulcent tonics and a strictly regulated diet,—and allaying nervous irritability and procuring sleep by means of night-draughts containing the acetate of morphia.

"A few days ago he felt some uneasiness in the right side: the cupping-glasses were had recourse to, but as he could not endure them, leeches were applied in their stead, and with a very good effect. He is now recovering rapidly."

Our readers are probably aware that a much more active method is adopted now-a-days, in the treatment of nepatic abscesses, than was deemed safe or warrantable a few years ago. Dr. Webb is a zealous advocate of the practice not only of early puncturing these abscesses and evacuating their contents as speedily as possible, but also of ascertaining the state of the liver by means of a *small exploring* trocar, as recommended by Dr. Murray, the late Deputy Inspector of Hospitals in India, Dr. Monat, and other surgeons of the Madras army, to whose skill and enterprize the profession is indebted for "this most important improvement in the treatment of hepatic abscess."

Before alluding to it more particularly, let us briefly review the opinions of some of the old physicians on this point of practice. That Hippocrates was in the habit of opening abscesses of the liver, either with the knife or the cautery, is well known to the classical reader. His description of the disease and the mode of its treatment is altogether so graphic that many of our readers, we doubt not, will be pleased to have it presented to their notice.

*Quum in latere tuberculum* fit, tussis emergit dura, et dolor, et febris, et gravitas in latere incumbit, et dolor acutus in eodem semper loco urget, et sitis vehemens, et calidum potum aversatur, et in dolens latus decumbere non potest, sed in sanum. At ubi decumbit, velut lapis impendere ipsi videtur, et intumescit, et rubescit, et pedes intumescunt: hunc secato aut urito. Postea pus emittito usque ad decimum diem, linamento ex lino crudo indito. Decimo autem die, pure omni emisso, vinum et oleum tepida immittito, ut ne de repente resiccetur, et linamento et linteolo utitor; ubi vero id quod infusum est emiseris, aliud: atque hæc per dies quinquæ facito. Postquam autem pus tenue effluxerit, velut ptisanæ ac modicum ad manus contactum, stanneum penicillum indito; et, ubi penitus resiccatum fuerit, linamento semper modice præsecto, ulcus ad linamentum coalescere sinito.—De Morbis, Lib.* II.

Aretæus (A.D. 81) recommends that an hepatic abscess should be opened with a red-hot knife, which *et secat et comburit.*†

This practice Baron Larrey followed on more than one occasion in his Egyptian Campaign with success.‡ He probably found it in use among the Arabs. It still exists among the native doctors in India.

Celsus says that, "Abscess in the liver is to be treated like other internal suppurations. Some open it with a scalpel and cauterise the vomica."

Galen (who died A.D. 200) introduced the digesting process, which was afterwards so generally practised in Europe; he condemns opening abscesses of the liver very early—*non expedit incidere protinus*; *sed digestionem moliri medicamentis ad id valentibus.*§

Hali Abbas (whom we have already quoted) lays down precise directions for opening hepatic abscesses with the cautery-knife; and Albucasis (who lived about a century later) has given the following description of the mode of performing the operation:—

"*Quando acciderit in jecore tumor, et scire cupias si is tumor fuerit in carne jecoris, vel in tunica ejus: equidem si fuerit in carne jecoris, invenies infirmum gravitatis sensu, et dolore minime acuto laborantem. Quodsi sit in tunica jecoris, cum dolore erit acuitas fortis, (dolor erit acutior valde:) Porro, si videris, quod curatio ejus fatigavit medicos, oportet ut infirmus decumbat in posticam cervicis partem (resupinus); tum signabit locum Apostematis cum atramento; dein calefac cauterium specillo simile, et hæc est ejus forma;* (a drawing of the instrument is

---

* *Phyma* (φυμα) *tuberculum,* generaliter dicitur omnis tumor p. n. erumpens; comprehendit igitur abscessus.—*Castelli Lexicon.*

† Medicæ Artis princip. H. Stephani, 1567, Lib. I. p. 32.

‡ Clinique Chirurgicale, tom. ii. p. 448-9.

§ Method. Medendi. Lib. XIII.

here given)—*et uras cum illo una ustione, donec cutis tota usta sit, et cum cauterio perveneris usque ad membranam adeo ut exire facias omnem materiem purulentam. Postea curabis curatione abscessuum donec sanetur.*"* 116.

In Van Swieten's Commentaries on the aphorisms of Boerhave, we read thus :—" the tumour of the liver, properly held or secured, is to be opened either by seton, actual cautery, caustics, or the lancet; and the wound made is to be afterwards gently corroded or enlarged to a greater depth by suppuratives and escarotics, until it extends to the vomica or abscess." .......... " But since delays are dangerous in the present malady, an incision is evidently to be preferred to the caustic."

The trocar appears to have been first used for the opening of hepatic abscesses in the latter half of last century. Vogel† and Benjamin Bell‡ are amongst the earliest authors who recommend it.

Of recent years, M. Recamier and other French practitioners have preferred the use of the caustic potash to that of the knife or trocar for the purpose alluded to; while Dr. Graves of Dublin has very ingeniously suggested,—with the view of inducing an adhesion between the diseased viscus and the abdominal parietes, and of preventing the risk of extravasation into the abdomen—that an incision should be made over the most prominent part of the tumour, and carried through the abdominal muscles, so as to reach without dividing, the peritoneum: by the subsequent application of poultices, &c. the tendency of the matter to come to the surface is rapidly promoted.

This method of treatment is doubtless too slow for the exigencies of East India practice, in which, unless prompt and almost immediate relief be given, the patient will too often rapidly sink.

We shall now adduce the reports of two or three cases of Hepatic Abscess detailed in Dr. Webb's interesting collection, with the view of illustrating some of the points to which we have alluded.

*Case* 1.—A middle-aged soldier, who had been 14 years in India, was received on the 21st March, 1839, into the hospital with symptoms of acute hepatitis. On the 23rd he had cold shiverings, followed by profuse sweats. On the 30th, Dr. Murray, suspecting the existence of an abscess, introduced a trocar, to the depth of an inch and a half, at the lateral part of the thoracic arch, between the eighth and ninth ribs. *About an ounce of very black blood flowed out by the canula, but without any admixture of pus.* A second puncture was made a little more anteriorly and deep, under the cartilage of the eighth rib: *this gave exit to more black blood,* and afforded great ease; but *still no pus flowed.* Tents of lint were introduced into the wounds, and large poultices applied over the side. The symptoms of suppuration continuing, Mr. Wilkins, the regimental surgeon, " having (we copy his report) a strong idea that the common trocar, had been too short, on the 5th April *introduced the one for puncturing the bladder by the rectum.*

---

* *Channing's* Albucasis, Oxonii 1778, p. 61, vol. i., Sect. xxviii, de tumore jecoris cauterio perforando.

† Prelectiones de cognoscendis Morbis. Edit. Gotting. 1772, vol. i. p. 176.

‡ System of Surgery, vol. v. p. 393, 5th edition.

which, being curved and longer than the other, reached the abscess, and gave vent to a quantity of sanious purulent matter, (about four ounces), to the great delight and relief of the patient sufferer. *On the* 11*th the long trocar was again introduced* as the discharge had stopped—the punctures were kept open by tents—antimonials, and hydriodate of potass were administered—the discharge soon became healthy pus, which gradually lessened in quantity until it finally ceased on the 10th May, when the wounds became so contracted that the tents could no longer enter, and soon finally closed. The liver resumed nearly its natural size, pressure could be borne without giving pain, the patient's health gradually improved, and he was discharged on the 7th of June; since which time he has been in the performance of all his military duties (now two months), without having required to come back once to the hospital for medicine."*

*Case* 2.—A soldier, æt. 33, had for about a month been affected with cough, dyspnœa and hectic fever, accompanied with obscure symptoms of Hepatic suppuration. "The right *rectus* muscle was more tense than the left, or rather it became so on attempting to examine the tumour, as if to screen it from pressure, which Mr. Twining gives as a characteristic symptom of central abscess of the liver." Such being the case, Dr. Murray resolved (March 2nd) to ascertain the state of the organ by puncturing the most prominent part of the fullness with a trocar: but nothing save a little blood flowed out when the stiletto was withdrawn. "Not satisfied with this exploration, I pushed the new exploratory instrument into the Liver, behind the middle of the side, between the 8th and 9th ribs, when, to our satisfaction, pus flowed: not however through the tube of the instrument, but by the side of it—apparently from my having gone beyond the abscess.

"I then withdrew the explorer, and introduced a large sized flat trocar, by which eight or nine ounces of thick curdy pus were evacuated. When the evacuation was nearly completed, a gurgling of air took place through the canula, apparently from the action of the Diaphragm, and a cork was then fitted to the canula (which was retained *in situ*) with directions to take it out at mid-day and in the evening, to allow accumulated matter to escape. A bit of sticking-plaster was applied over the orifice of the first puncture in the Epigastrium."

The symptoms were considerably relieved by the operation for two or three days. On the 5th, "Dr. Murray made another exploratory puncture into the liver on the right side of the Epigastrium, without finding pus; but a quantity of serous fluid was evacuated from the cavity of the abdomen, on withdrawing the canula out of the Liver." He died on the 13th.

*Dissection.*—Before opening the body, the exploratory instrument was pushed into the liver near the end of the eleventh rib, when thin yellow pus issued freely through its canal, shewing it had entered an abscess. When the abdominal parietes were divided, the liver was found to be enormously enlarged: it adhered firmly, by recently-effused lymph, to

* Madras Quarterly Medical Journal, Vol. 2.

them, where the two punctures had been made in the epigastric region, nor were there any signs of inflammation of its parenchyma where the trocar had entered. "It was found that the exploratory instrument, which before dissection was pushed into the liver, had entered a large distinct abscess situated in the right side of the concave surface of the gland, which had very narrowly escaped being penetrated in the exploration made on the 5th instant. Its area was considerably larger than a man's fist, and it contained upwards of a pint and a half of thick yellow greenish pus.

"Immediately above this abscess, was the empty contracted sac of the one opened and evacuated on the 2nd March: the inner surface of it had a dark gangrenous appearance, which extended throughout the course of the wound.

"At the centre of the upper convex part of the liver was a third distinct abscess, *the largest of all*, containing nearly 3 pints of matter, which seemed not only the chief cause of the projection of the organ beyond the ribs, by its pushing it downwards; but also of the projection of the diaphragm into the right cavity of the chest: it pushed the diaphragm as high up as the 4th rib. The upper part of the walls of this abscess adhered extensively to the diaphragm; as did those of the lowest abscess to the cellular substance and other parts above the right kidney."

In the clinical remarks which Inspector Murray appends to the history of this case, he observes:—

"After having seen this dissection, I would hereafter explore to a greater extent any analogous case; and I am, moreover, of opinion, that all our punctures should be made from the abdominal cavity—entering the trocar or explorer under the edge of the cartilages of the 7th, 8th, or 9th ribs, as circumstances may indicate. We may often indeed get nearer to the abscess through one of the intercostal spaces; *and I think primary exploration may sometimes be advantageously made in this situation by a very minute flat canular instrument;* but, from not having seen any patient recover where the matter was evacuated in this direction (through the diaphragm); from finding that the action of the fibres of the diaphragm impedes the free discharge of the matter, somewhat like a valve; from observing that air sometimes enters the wound when made here; and from considering that the opening is not so dependent through the walls of the thorax as when made through the abdominal parietes; I beg to recommend the latter mode *in all cases;* and I must also say that I would prefer a long flat trocar to any other instrument, as the stilette can be withdrawn occasionally during the operation to ascertain if any abscess has been penetrated; and the canula can be left in *situ* afterwards, if thought desirable." 102.*

In another most unfavourable case, Dr. M. drew off more than *nine pints* of thin flaky purulent matter: the canula was left in the liver with the view of allowing the matter to drain off as it formed. The operation was more successful in the case related by our author in a subsequent page (130): on that occasion, about a pint and a half of thin brown matter came out on withdrawing the stilette, and the canula was left in the wound.

---

* Madras Quarterly Medical Journal, vol 2, p. 232.

In the following one, a cure was effected by the use of the seton.

*Case.*—A soldier, æt. 24, was seized with all the symptoms of acute Hepatitis, on the 14th July, 1839. Although the active symptoms were subdued by antiphlogistic treatment, an abscess eventually formed in the liver. On the 13th of August, (a month from the date of his admission into the hospital,) an incision was made down to the peritoneum, an inch and a half below the margin of the ribs, and a seton inserted. In the course of a few days, a copious thick, green, and fœtid purulent discharge escaped from the side of the seton, and the enlargement of the liver was very manifestly diminished. On the 27th, the report states that there was a slight natural discharge from the seton, and that the size of the liver was nearly reduced to its normal condition. In other three weeks, the patient was declared convalescent. The following practical remarks are appended by the narrator, Dr. John Murray, to the preceding history.*

"In this case I am confident abscess existed before the patient's last admission; and had the swelling not been so near the situation of the gall-bladder, a preparatory incision down to the peritoneum would have been made on the 17th of July; but having seen a distended gall-bladder as large as the tumour then was, and finding that it diminished by the means used, I was induced to defer the operation. Irregularity in diet caused a relapse. After which there was no doubt in the diagnosis; but as the effect of internal remedies had been so satisfactory in the first instance, they were again tried. After I made the incision into the integuments and inserted the seton for the purpose of bringing on adhesion between the liver and peritoneum, the tumour again diminished in such a manner as to induce me to delay making any puncture; and on the afternoon of the 27th August the abscess burst through the part where the incision was made, and where adhesion had evidently taken place; after which the patient's restoration to health has been progressive, and I have no doubt of a favourable termination.

"The abscess should have been punctured on the 15th or 16th of August; and though the delay led to no unfavourable consequences in this case, I by no means recommend my example to be followed; for when the walls of the sac become thin, there is no knowing when and where it may give way, and if the abscess had burst into the cavity of the peritoneum, the patient would have inevitably died, and I should have been to blame for indecision in my practice, after the light which has lately been thrown on this subject." 130.

We take leave of Dr. Webb with the highest esteem for his talents, and look forward with pleasure to the appearance of the second part of his Pathologia Indica. It contains more valuable matter in its small compass than a dozen of the ordinary run of medical works, that now-a-days issue from the press in this country.

---

* Madras Journal, Vol. III. p. 275.

